# Characteristics of Dissolved Organic Matter in Sediments of Typical Lakes in Southeastern Hubei Province, China

**DOI:** 10.3390/ijerph19127402

**Published:** 2022-06-16

**Authors:** Chao Wu, Xiaodong Wu, Xuguang Ge, Lian Feng, Ya Tan, Jiuyun Yang, Weixiang Ren, Min Zou

**Affiliations:** 1 College of Urban and Environmental Sciences, Hubei Normal University, Huangshi 435002, China; wuchao9905@163.com (C.W.); gxg76@hbnu.edu.cn (X.G.); afenglian@163.com (L.F.); 17347795556@163.com (Y.T.); v1572722@126.com (J.Y.); a17282394244@163.com (M.Z.); 2 China Aero Geophysical Survey & Remote Sensing Center for Natural Resources, Beijing 100083, China; daxian_ren@163.com; 3 School of Earth Sciences and Resources, China University of Geosciences, Beijing 100083, China

**Keywords:** southeastern Hubei Province, China, lake, sediment, dissolved organic matter

## Abstract

This study aimed to reveal the distribution characteristics and sources of dissolved organic matter (DOM) in sediments of typical lakes in the southeastern of the Hubei province and provide a reference for pollution control of eutrophic lakes. The sediments of four typical lakes (Liangzi, Baoan, Daye, and Qingshan) in the southeastern of the Hubei Province were investigated in May 2019. Ultraviolet-visible (UV-Vis) absorption spectroscopy and excitation-emission matrixes characteristics of DOM were obtained by UV-Vis spectrophotometry and three-dimensional fluorescence spectroscopy. Moreover, the DOM fluorescence components were resolved by parallel factor analysis (PARAFAC). The results showed that: (1) The absorption coefficient *a*_350_ in surface sediments followed the order: Baoan Lake (16.99 ± 3.82 m^−1^) > Qingshan Lake (15.37 ± 3.8 m^−1^) > Liangzi Lake (12.54 ± 5.58 m^−1^) > Daye Lake (12.43 ± 1.43 m^−1^). Specifically, with increasing depth in the sediment cores, *a*_350_ increased and then gradually decreased in Daye Lake and Baoan Lake, but fluctuated in Liangzi Lake and Qingshan Lake with a roughly decreasing trend. (2) Two humic-like components (C1, C2) and one protein-like component (C3) were identified via PARAFAC. This analysis also showed that the surface sediment fluorescent dissolved organic matter (FDOM) was dominated by protein-like, while the sediment core FDOM was dominated by humic-like in Liangzi Lake. The proportion of humic-like in FDOM was higher than protein-like in surface sediments and sediment cores of Baoan, Daye, and Qingshan Lakes. (3) The DOM of surface sediments in the Liangzi Lake comprised mostly autochthonous components, mainly produced by the vital activities of aquatic organisms. The DOM of surface sediments in Baoan Lake and Qingshan Lake showed weak humic and moderate autochthonous characteristics. The humification of DOM in sediment cores of Liangzi, Baoan, and Qingshan Lakes gradually decreased from depth to the surface. The DOM in surface sediments of Daye Lake had strong humic and moderate autochthonous characteristics. (4) In general, the DOM of lake sediments in southeastern Hubei Province has dual-contribution characteristics representing terrigenous and endogenous sources. In the restoration and treatment of lake ecology, both internal and terrestrial pollution should be considered. At the same time, it is noteworthy that cyanobacteria depletion and aquatic residues caused potential internal pollution.

## 1. Introduction

Dissolved organic matter (DOM) is ubiquitous in water, soils, and sediments. It constitutes one of the active organic components in nature and plays a vital role in mass-circulation within and among ecological systems [1,2]. DOM in lakes has complex compositions originating from either endogenous production or terrigenous inputs [3]. When exogenous pollution in lakes is effectively controlled, the effects of endogenous pollution will become more evident, intensifying the eutrophication of lakes [4]. Researchers have gradually shifted their attention to sediments to better understand endogenous phenomena within lakes to better understand endogenous phenomena. DOM in lake sediments serves as a carrier for various pollutants, and its mineralization processes release nutrients (nitrogen and phosphorous) to the overlying water, and consume oxygen from the water, thus impacting the nutrient and oxygen concentrations balance in lakes [5].

The existing research on DOM has focused more on its sources, components, environmental significance, etc. [6,7,8]. As an important sediments’ component of sediments, DOM can affect various processes, such as heavy metal migration, nutrient cycling, microbial growth, etc. Gao et al. [9] found that DOM had different effects on the migration of heavy metals copper, cadmium, lead, and zinc in soil. In the Baiyangdian Lake, it was documented that protein-like substances produced by the decay of submerged plant residues showed more robust and stronger complexation capability towards heavy metal ions compared to humic acid-like components [10]. Mercury cycling and microbial communities in sediments of oligotrophic wetlands were also shown to be affected by DOM inputs [11]. DOM fluorescence components of DOM can, to some extent, indicate the eutrophication degree of eutrophication in lakes [12]. Components of DOM vary dramatically depending on their location and surrounding environments. DOM in the sediments of Poyang Lake was mainly imported from terrestrial sources [13]. DOM in the sediments of Songhua Lake in Jilin Province was mainly terrigenous, and the fluorescence intensity of each component in the upstream was higher than that in the downstream [14]. DOM in the sediments of the eutrophic Chaohu Lake were primarily produced by the biological activities of organisms (e.g., algae) in the water [15], while DOM in the sediments of Erhai Lake sediments consisted mostly of protein-like and humus-like substances [16]. DOM in the interstitial water of the sediments in typical waters of the Baiyangdian Lake had strong autochthonous but weak humification characteristics [17]. DOM in the sediments of the Gangnan Reservoir in Hebei province also showed weak humification and strong authigenic characteristics [18].

With the advancement of instrumental analysis technology, the three-dimensional fluorescence spectroscopy technique (3D-EEMs) has become a highly efficient and convenient method for the semi-quantitative analysis of DOM. It is easy to operate, highly sensitive, and does not destroy the organics’ components, thus giving it broader applicability to various studies [3,19].

In recent years, the lakes in the southeastern region of the Hubei Province in China have been subjected to pollution at different levels, but many relevant issues have been left unexplored. Therefore, in this study, four typical lakes in the southeastern of the Hubei, China, were selected as research objects to reveal the distribution characteristics and sources of DOM in sediments using a combination of methods including parallel factor analysis (PARAFAC) and ultraviolet-visible (UV-Vis) spectrophotometry.

## 2. Materials and Methods

### 2.1. Overview of the Study Area

Four typical lakes in the southeastern part of the Hubei Province, China (113°31′52.356″ E~116°7′53.688″ E, 29°1′52.140″ N~30°53′57.012″ N) were selected as research objects based on the environmental significance of DOM (Table 1). Specifically, Liangzi Lake, Baoan Lake, and Qingshan Lake have macrophytic, macrophytic-to-algal, and urban algal features, respectively, while Daye Lake is an urban-suburban algal lake with serious terrestrial pollution sources. The surface area of Liangzi lake, which experiences less industrial pollution, is 271.00 km^2^ [20]. The Baoan Lake surface area is 45.10 km^2^, with east-west width of 7.50 km and a south-north length of 11.50 km; *Potamogetom crispus* L. flourishes in spring, but dies in summer and is succeeded by no submerged plants; thus leading to a macrophytic-to-algal transformation [20]. Daye Lake is located southeast of Daye City with a surface area of 54.70 km^2^ and 28 tributaries to the basin, and it receives heavy industrial pollution [20]. Qingshan Lake is in Huangshi City, has a surface area of 0.15 km^2^, and is a typical urban eutrophic lake [21].

### 2.2. Sample Collection and Index Determination

**Figure 1:**
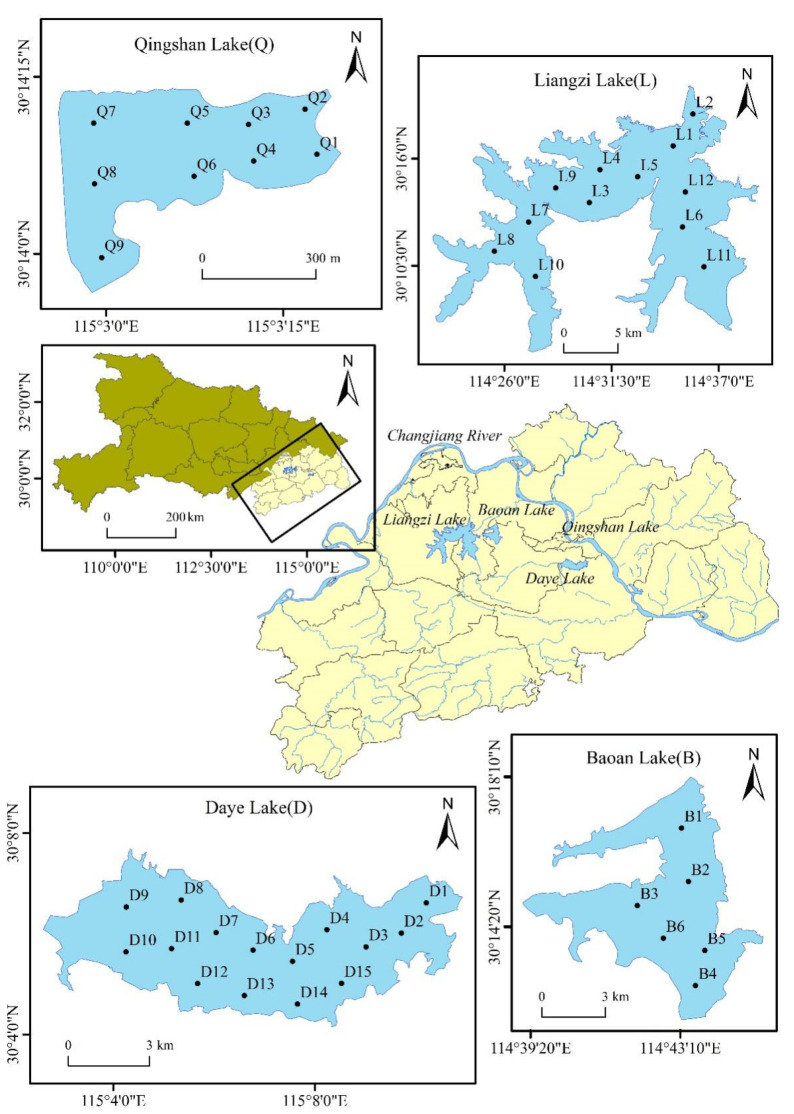
Locations of the typical lakes in southeastern Hubei and sampling points.

In spring 2019, surface sediment and surface water samples were collected from each lake using the Peterson sediment sampler and a Plexiglas water picker (Figure 1). Two representative positions (Table 1) were selected in each lake to collect sediment core samples (0 to 40 cm) using a column sediment sampler. Sediment cores were immediately divided into 2 cm thick sections upon transport back to the lab. All sediment samples were freeze-dried, ground, and sieved through a 100-mesh sieve. The dry sediment samples were extracted in Milli-Q ultrapure water (solid to water ratio of 1:10, *w*/*v*) while shaking for 24 h (25 °C, 220 r/min). The resulting mixture was centrifuged for 10 min (10,000 r/min), and the supernatant was filtered through a glass fiber filter (pore size: 0.45 μm) to collect the filtrate for further analysis [22].

In-situ water depth and transparency measurements were performed using a bathymetry meter and a Secchi disc. The physicochemical indexes were determined with reference to the Analytical Methods for Water and Wastewater Monitoring (4th Ed.) [23].

### 2.3. Determination of DOM by UV-Vis Spectroscopy

The filtrate was scanned by a UV-Vis spectrophotometer (UV2700; Shimadzu, Kyoto, Japan) to obtain UV-Vis data. Milli-Q ultrapure water was used as a reference, and the scan covered the wavelengths from 200 to 800 nm at an interval of 1 nm. The spectral scanning rate was 300 nm/min. The following equations were adopted to calculate the uncorrected and corrected absorption coefficients [24,25]:

*a_λ_*’ = 2.303 × *A_λ_*/*r* (1)

*a_λ_* = *a_λ_*’ − *a*_700_’ × *λ*/700 (2)

where *a_λ_*’ (unit: m^−1^) is the uncorrected absorption coefficient at wavelength *λ* (unit: nm); *A_λ_* is the absorbance at wavelength *λ* (denoted by Abs), and *r* is the optical path of the cuvette (unit: m). Equation (2) was utilized for scattering correction, where *a_λ_* (unit: m^−1^) is the corrected absorption coefficient at wavelength *λ* (unit: nm).

The absorption coefficient ratio (M) was also calculated from UV-Vis, and defined as the ratio of absorption coefficients of DOM at the 250 nm and 365 nm wavelengths.

### 2.4. 3D-EEMs Measurement of DOM and PARAFAC

A fluorescence spectrophotometer (Fluorolog-3; HORIBA, Kyoto, Japan) with Milli-Q ultrapure water as a blank was used to conduct the 3D-EEMs scanning of DOM. The excitation (Ex) wavelength ranges from 250~450 nm with 5 nm increments, and the emission (Em) wavelength ranges from 250~580 nm with an increment of 1 nm [25]. The slit width of the excitation and emission monochromators was 5 nm, and the integration time was 0.2 s [25]. The drEEM and N-way toolkit were used for PARAFAC in Matlab to determine the fluorescent dissolved organic matter (FDOM) components. To validate the model, a split-half analysis was performed to determine the optimal components before guaranteeing the removal of outliers. Split-half analysis tests show that the final three-component model can interpret the total EEMs variables. The maximum fluorescence intensity (F_max_) of each component obtained by PARAFAC was used to represent the actual concentration of the corresponding FDOM component. The results were compared with models from the online database (http://www.openfluor.org/ 21 April 2021) to determine the characteristics of each component. Components were considered a match when the respective similarities between excitation and emission spectra were higher than 94% [26,27].

The humification index (HIX), biogenic index (BIX), and fluorescence index (FI) were obtained via 3D-EEMs. HIX refers to the ratio of the integral of the fluorescence value obtained with Em at 435–480 nm to that at 300–345 nm when the Ex was fixed at 254 nm. BIX referred to the fluorescence intensity ratio of Em at 380 nm and 430 nm when Ex was fixed at 310 nm. FI refers to the fluorescence intensities ratio of Em at 470 nm and 520 nm when Ex was fixed at 370 nm.

### 2.5. Statistical Analysis

ArcGIS 10.7 (ESRI, Redlands, CA, USA) was used to draw the distribution map of sampling points and perform further spatial interpolation analysis. The SPSS 21 (IBM, New York, NY, USA) was used to analyze the obtained data and statistical figures were drawn with the Origin 2019. As for significance level, *p* < 0.01 was considered highly significant, 0.01 ≤ *p* < 0.05 was considered significant, and *p* ≥ 0.05 was considered insignificant.

## 3. Results

### 3.1. Physiochemical Indicators of Water Bodies and Surface Sediments

As shown in Table 2, the nutrient levels of four typical lakes in southeastern Hubei were different. The total nitrogen (TN), total phosphorous (TP), and the permanganate index (COD_Mn_) of water exhibited identical patterns among the four lakes, with the highest levels in the Qingshan Lake, followed by Daye, Baoan, and Liangzi lakes. The highest TN in surface sediment was found in the Baoan Lake (1257.54 ± 313.29 mg/kg), significantly higher than the Liangzi Lake (*p* < 0.05). The highest and lowest TP concentrations of surface sediments were found in the Qingshan (1201.77 ± 320.50 mg/kg) and Liangzi Lakes (420.63 ± 106.99 mg/kg), respectively. The highest and lowest organic matter (OM) concentrations of surface sediments were found in the Qingshan Lake (71.32 ± 19.09 mg/kg) and the Daye Lake (36.15 ± 10.73 mg/kg), respectively.

### 3.2. UV-Vis Distribution Characteristics

#### 3.2.1. UV-Vis Distribution Characteristics of DOM in Surface Sediments

DOM Absorption coefficients *a*_254_, *a*_280_ and *a*_350_ are widely used to describe the abundance of DOM in the ecosystem. In this work, *a*_350_ was selected to characterize the relative concentrations of DOM [28,29]. The DOM *a*_350_ values in the surface sediments revealed clear spatial differences in the four lakes (Supplementary Figure S1a–d). The maximum *a*_350_ was found in the Baoan Lake (16.99 ± 3.82 m^−1^), followed by the Qingshan Lake (15.37 ± 3.82 m^−1^), the Liangzi Lake (12.54 ± 5.58 m^−1^), and the Daye Lake (12.43 ± 1.43 m^−1^).

As shown in Supplementary Figure S2, the M values of surface sediments in the four lakes were highly significantly different (*p* < 0.01). They followed the order: Baoan Lake (5.87 ± 0.56) > Qingshan Lake (5.05 ± 0.80) > Daye Lake (4.98 ± 0.62) > Liangzi Lake (3.97 ± 0.71). In Liangzi Lake the maximum surface sediment M value (5.25) occurred at L11, but the maximum at Baoan Lake occurred at B1 (6.80) in the north and the maximum at Qingshan Lake occurred at Q6 (6.70).

#### 3.2.2. Vertical Distributions of UV-Vis of DOM in Sediment Cores

The vertical distribution characteristics of absorbance coefficients *a*_350_ of DOM in sediment cores of Liangzi Lake, Baoan Lake, and Qingshan Lake were highly significantly different (*p* < 0.01), as seen in Supplementary Figure S3. The mean *a*_350_ value in the sediment core of Qingshan Lake was 20.85 ± 6.97 m^−1^, significantly higher than those of Liangzi Lake and Baoan Lake (*p* < 0.01). The *a*_350_ distributions in the sediment cores of Daye Lake and Baoan Lake were similar, initially increasing with depth, but then gradually decreasing. However, the *a*_350_ vertical distributions in Liangzi Lake and Qingshan Lake were different, showing a general decreasing trend with increasing sediment cores depth.

With increasing depth in the sediment core, the sediment M value in the Liangzi Lake gradually increased until peaking at 16 cm (8.23 ± 3.67), after which it decreased before stabilizing between 4.17–6.12 after 22 cm deep (Supplementary Figure S4a). Supplementary Figure S4b shows a similar tendency in Baoan Lake, but with the maximum M value occurring at 18 cm (12.80 ± 3.01). With the increasing depth in the sediment core, the sediment M value in Daye Lake fluctuated sharply (Supplementary Figure S4c), ranging from 4.48 to 24.03, with an average value was 12.33 ± 7.63. With increasing sediment core depth in Qingshan Lake, M peaked at 10 cm (9.96 ± 2.49), followed by a decrease before fluctuating between 4.32–7.43 after 14 cm (Supplementary Figure S4d).

### 3.3. Characteristics of FDOM Components

**Figure 2:**
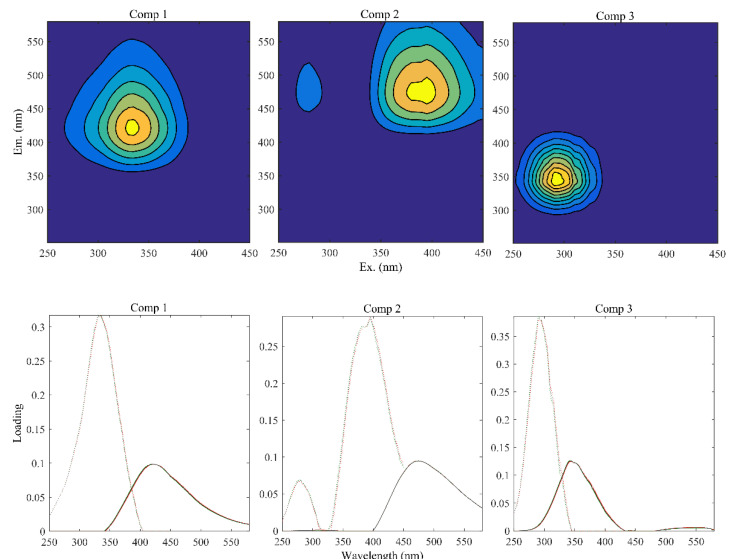
Fluorescent components were separated using PARAFAC and their split-half analysis tests.

The PARAFAC model, validated by split-half analysis, identified three fluorescent components of FDOM (Figure 2). The upper images in Figure 2 showed the location of the fluorescence peak. The lower images in Figure 2 correspond to split-half analysis results for each component. The curves tested in each component almost overlap, indicating that the components were very similar in shape and could almost well verify the fluorescence characteristics. The obtained components were compared to the published OpenFluor online database to acquire the characteristics of each, as shown in Table 3.

Component 1 (C1) showed a maximum fluorescence peak at Ex = 335 nm and Em = 422 nm, located near the C peak of the conventional peak group, usually considered a humus-like component in the visible zone. C1 is generally related to human activities (e.g., agricultural practices) and microbial degradation, and is found in wastewater and eutrophic water bodies [25,30,31,32]. Component 2 (C2) featured two peak excitation wavelengths (280 nm and 395 nm) at Em = 473 nm, located near peaks A and D in the conventional peak group, respectively. Similar studies have found close matches to this component, and have tentatively identified it as a terrigenous humus-like component in the UV zone and soil fulvic acid; however, the component C2 exact sources require further exploration. The maximum Ex and Em of component 3 (C3) were at 290 nm and 344 nm, respectively, located near peak T in the conventional peak group. The main component was autochthonous and protein-like (tryptophan-like), and mostly related to the endogenous production by phytoplankton and microbial communities [33,34,35,36].

**Figure 3:**
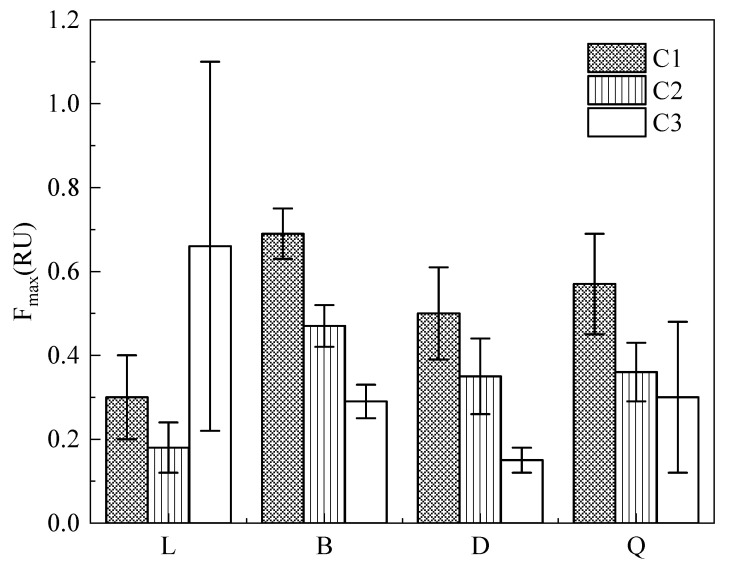
Proportions of FDOM components in surface sediments of four typical lakes.

The maximum intensities of the three fluorescent components were recorded as F_1max_, F_2max_, and F_3max_. The C1 and C2 components in surface sediments shared identical patterns among lakes (Figure 3), with the order: Baoan Lake > Qingshan Lake > Daye Lake > Liangzi Lake. The surface sediment F_1max_ mean value in Baoan Lake was 0.69 ± 0.06 RU (Raman Unit; RU), significantly higher than Qingshan Lake (*p* < 0.05). The F_2max_ values of surface sediments from the four lakes showed highly significant differences (*p* < 0.01). Liangzi Lake had the lowest F_2max_ (0.18 ± 0.06 RU) in its surface sediments, significantly lower than Baoan Lake, Daye Lake, and Qingshan Lake (*p* < 0.01). The maximum and minimum F_3max_ values occurred in Liangzi Lake (0.66 ± 0.44 RU) and Daye Lake (0.15 ± 0.03 RU).

As indicated in Supplementary Table S1, the F_1max_ values in the sediment cores from the four lakes had highly significant differences (*p* < 0.01). The vertical distribution revealed that F_1max_ had identical vertical distribution patterns in Baoan Lake and Daye Lake, with gradual decreases with increasing depth in the sediment cores. The overall averaged F_2max_ values for the sediment cores from the four lakes were ordered as follows: Liangzi Lake (0.14 ± 0.04 RU) < Daye Lake (0.27 ± 0.16 RU) < Baoan Lake (0.33 ± 0.09 RU) < Qingshan Lake (0.47 ± 0.15 RU), and there were highly significant differences among Liangzi Lake, Baoan Lake, and Qingshan Lake (*p* < 0.01). The F_3max_ values showed highly significant differences among Liangzi Lake, Baoan Lake, and Daye Lake (*p* < 0.01) (Supplementary Table S1). The three components shared identical vertical distribution patterns in the sediment cores of Daye Lake; that is, their fluorescence intensities all gradually decreased with increasing depth.

## 4. Discussion

### 4.1. Analysis of FDOM Source

**Figure 4:**
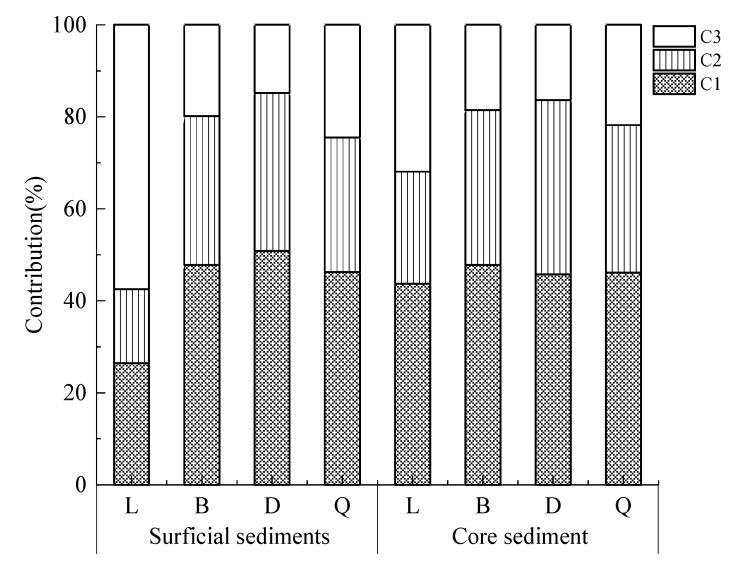
Contribution of fluorescent components in sediments to FDOM.

Humus-like components are primarily composed of exogenous inputs, while protein-like components mainly comprise endogenous sources [43]. In this study, C1 and C2 were identified as terrigenous components, with C1 being most closely correlated to agricultural activities; C3 was identified as an endogenous component. Previous studies have indicated that increasing intensities of agricultural activities around lakes leads to increased input of terrigenous humus-like substances [44,45]. This was reflected in Baoan Lake and Daye Lake, which are surrounded by widely distributed agricultural areas, and whose total proportions of component C1 in the FDOM of sediments were 47.82% and 47.41%, respectively (Figure 4). Although agricultural activities around the Liangzi Lake were also ongoing, the intensity had diminished due to strengthened watershed management; its C1 component only accounted for 26.42% of the FDOM of surface sediment (Figure 4). The land in the Daye Lake watershed was mainly used as arable land, which accounted for 47.60% [46]. The numerous rivers in the Daye Lake watershed pick up large amounts of terrigenous humus when flowing through arable areas before entering the lake, especially when the runoff volume increase, thus increasing the proportion of C1 in FDOM. Since the 1980s, industrial wastewater from enterprises (e.g., mines) and domestic sewage have been discharged arbitrarily without treatment before flowing into lakes, giving rise to serious terrigenous pollution in Daye Lake [20]. In this study, humus-like C1 and C2 were the main contributors to the sediment FDOM in the Baoan Lake, with 81.18% (Figure 4). This was consistent with the findings of Ren et al. [47], showing that humus-like components were also the main contributors to the water FDOM in Baoan Lake. Du et al. [48] pointed out that urban domestic sewage and aquaculture wastewater constituted the main sources of anthropogenic FDOM. Qingshan Lake is located in the old town of Huangshi City, which has not established rainwater and sewage diversion infrastructure due to historical restraints. Furthermore, the wastewater treatment plant capacity on the west side of Qingshan Lake was limited, so a small amount of domestic sewage was directly released into the lake without treatment. Similarly, upstream aquaculture fish ponds ultimately generate terrigenous organic matter that flows into the lake [49]. In addition, the concrete and asphalt pavements around Qingshan Lake reduced runoff infiltration, resulting in the direct input of terrigenous humus-like components from surface soils to the lake [44,50].

Eutrophication in lakes means abundant nutrients and carbon sources for bacteria and algae, resulting in the rapid growth of biological communities. Once the algal cells are degraded, and are turned into autochthonous DOM [29,51,52]. Algal blooms of varying scales have been observed in summer in Baoan Lake, Daye Lake, and Qingshan Lake; these directly contribute component C3 to the lakes. In addition, protein-like components have also been linked to the decay and degradation of aquatic plants [53]. In spring, the aquatic vegetation (e.g., *Potamogeton crispus* L.) in Liangzi Lake and Baoan Lake thrives, producing large amounts of protein-like components upon dying and being degraded and serving as another main source of C3.

The FDOM components in the sediments of the four lakes differed due to environmental differences. As shown in Figure 4, the main contributor to FDOM of the surface sediment in Liangzi Lake was C3 (57.50%), followed by C1 (26.42%) and C2 (16.80%). Overall, the main contributor to FDOM in the sediment cores in Liangzi Lake (Figure 4) was C1 (43.71%), followed by C3 (31.94%) and C2 (24.36%). The main contributor to FDOM in the surface sediments of Baoan Lake, Daye Lake, and Qingshan Lake was C1, followed by C2 and then C3 (Figure 4), which was also true throughout the FDOM vertical distributions in the sediment cores from the three lakes. These combined findings signified that terrigenous humus accounted for a higher proportion than autochthonous protein in the FDOM of sediments from these three lakes. With increasing sediment core depth, the fluorescence intensities of the three components (C1, C2, and C3) all decreased to a certain extent in Baoan Lake, Daye Lake, and Qingshan Lake (Supplementary Table S1), possibly due to the gradual decrease of microbial abundance and activity arising from gradually decreasing concentrations of nutrients with sediment depth [54].

Few studies can match component C2 well (Table 3); indeed, in this study, there was a highly significant correlation between C1 and C2 in all four lakes (*p* < 0.01), as shown in Supplementary Table S2. This was in line with the results of Zhai et al. [54] at Yinghu Lake where C1 appeared to have some relationship with C2. Overall, the absorbance coefficient *a*_350_ in the DOM of the sediments from the four lakes was significantly correlated with the fluorescent intensity of each component (*p* < 0.05), indicating a close relationship between FDOM and DOM (Supplementary Table S2). In the Baoan Lake, Daye Lake, and Qingshan Lake sediments, the fluorescence intensity of the three components all had significant positive correlations with TN, TP, and OM (*p* < 0.05), indicating that the FDOM in the sediments of the three lakes were also closely related with the migration of nutrients such as N and P (Supplementary Table S2). Therefore, the measured fluorescence intensity of the components in FDOM can, to some extent, indirectly reflect the organic pollutants distributions in lake sediments [48].

### 4.2. Differences in Biogeochemical Characteristics of DOM among Sediments of Four Typical Lakes in Southeastern Hubei

**Figure 5:**
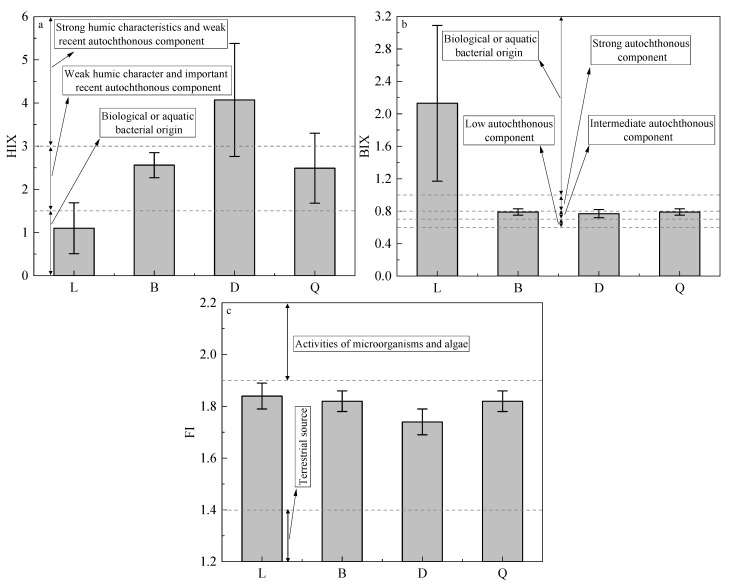
Distributions of HIX (**a**), BIX (**b**), and FI (**c**) in surface sediments of four typical lakes.

When 0 < HIX < 1.5, DOM undergoes a low degree of humification and is mainly produced by endogenous organisms and bacteria; when 1.5 < HIX < 3, DOM is characterized by weak humification from recent autochthonous sources; when 3 < HIX < 6, DOM has strong humification characteristics with high terrigenous contributions [55]. The lowest HIX values were found in the surface sediment of Liangzi Lake, with a mean of 1.10 ± 0.59, indicating the DOM was mainly autochthonous (Figure 5a). This was confirmed by the FDOM of the surface sediment of Liangzi Lake, in which the autochthonous protein-like component C3 was the main contributor (Figure 4). The mean HIX values in the surface sediments of Baoan Lake and Qingshan Lake were 2.56 ± 0.29 and 2.49 ± 0.81, respectively, with a combined range of 1.5–3; therefore, the DOM in these two lakes had weak humification and important recent autochthonous sources, the latter of which may be the decay and degradation of *Potamogeton crispus* L. and cyanobacteria (Figure 5a). The surface sediment of Daye Lake showed the highest mean HIX value of 4.07 ± 1.31 (range of 3–6), indicating the DOM had high humification characteristics (Figure 5a), consistent with the patterns observed in the FDOM components. The terrigenous humus-like components C1 and C2 accounted for a higher proportion (85.14%) of the FDOM of surface sediments in Daye Lake (Figure 4). The vertical distributions revealed a roughly decreasing trend in HIX values from the deepest layers to the surface of the sediment cores of Liangzi Lake, Baoan Lake, and Qingshan Lake (Supplementary Table S3). This suggested that the DOM in deep sediments had higher degrees of humification in these three lakes. The mean HIX values at different depths in the sediment core from Daye Lake all ranged from 3 to 6, pointing to strong humification of the DOM (Supplementary Table S3).

BIX reflects the relative contributions of autochthonous components to DOM. Typically, DOM have low, moderate, and strong autochthonous characteristics when 0.6 < BIX < 0.7, 0.7 < BIX < 0.8, and 0.8 < BIX < 1, respectively. When BIX > 1, the endogenous components produced by bacterial activity account for a very high proportion [56]. All the mean BIX values were greater than 1 in the DOM of surface and core sediments in Liangzi Lake, indicating that the vital activities of organisms and bacteria mainly produced the DOM endogenous components in Liangzi Lake. Moreover, the endogenous production in Liangzi Lake was higher than in the other three lakes (Figure 5b and Supplementary Table S3). The mean BIX values of Baoan Lake, Daye Lake, and Qingshan Lake were 0.79 ± 0.04, 0.77 ± 0.05, and 0.79 ± 0.04, respectively (combined range of 0.7–0.8), indicating their DOM had moderate autochthonous characteristics (Figure 5b). The vertical distribution showed that the mean BIX values varied from 0.8 to 1 at different depths in the sediment cores from Baoan Lake, Daye Lake, and Qingshan Lake, indicating that the DOM had strong autochthonous characteristics (Supplementary Table S3). BIX gradually increased in Baoan Lake and Daye Lake with increasing depth in the sediment cores, suggesting increased relative contributions of autochthonous components to DOM (Supplementary Table S3).

FI can be used to identify the humus sources in DOM. Typically, when FI < 1.4, the primary contribution comes from terrigenous sources; when FI > 1.9, humus is mainly produced by microbial metabolic processes; and when 1.4 < FI < 1.9, there are dual-contribution characteristics from both terrigenous and endogenous sources [57,58]. All FI values in the surface and core sediments of the four lakes ranged from 1.4 to 1.9 (Figure 5c and Supplementary Table S3), indicating that the humus components in the DOM of sediments had dual-contribution characteristics representing terrigenous and endogenous sources.

The parameter M of the DOM UV-Vis spectra can be used to evaluate the molecular weight of DOM. Generally, humic acid is of higher molecular weight while fulvic acid is of lower molecular weight, so higher proportions of humic acid contribute to higher molecular weights of DOM, and consequently, lower M values [54,59,60]. When M > 3.5, there is a higher proportion of fulvic acid than humic acid in DOM, and vice versa when M < 3.5 [61]. Supplementary Figures S2 and S4 show that the mean M values in the surface and core sediments of the four lakes were all larger than 3.5, suggesting that the fulvic acid proportion is generally higher than humic acid in the DOM of sediments.

### 4.3. Spectral Parameters of DOM in Surface Sediments of Different Study Areas

The spectral parameters of the DOM in surface sediments of the different lakes are shown in Table 4. The HIX values of the surface sediment DOM of Daye Lake were close to those of Nanfei River, indicating their similarity in terms of terrigenous pollutants, which are largely affected by human activity. The Nanfei River is located in the main urban area of Hefei city, and there are many different types of outlets in this reach. A large amount of industrial wastewater and municipal sewage entered the Nanfei River through the drainage, resulting in serious land pollution. Daye Lake also has a similar situation, its west is the main urban area of Daye City, and some rivers into the lake water quality is poor. A large amount of industrial, agricultural, and domestic sewage entered the lake through runoff, resulting in serious land-based pollution of Daye Lake, which made the HIX of the DOM of its sediment similar to that of Nanfei River. The terrestrial pollution sources of Liangzi Lake, Baoan Lake, and Qingshan Lake are relatively light. Compared with Nanfei River, Danjiangkou Reservoir, Lihu Lake, Jinpen Reservoir, and Xiaojiajiang River, the mean HIX values of DOM in surface sediments of Liangzi Lake, Baoan Lake, and Qingshan Lake were generally lower. The DOM generated by the decomposition of aquatic plants and algae in lakes with different nutrient levels may be different, which may be an important reason for the difference in BIX. For BIX, however, previous research has found the mean BIX values of DOM in surface sediments to range from 0.7 to 0.8 (except for Nanfei River, Chaohu Lake, and Xiaojiajiang River), which was similar to the results from Baoan Lake, Daye Lake, and Qingshan Lake in this study. The mean FI values of DOM in surface sediments of Danjiangkou Reservoir, Jinpen Reservoir, and Arctic Kongsfjorden were all between 1.4–1.9, consistent with the surface sediments results from the four lakes in this study, where the humus in the DOM came from terrigenous and endogenous sources.

## 5. Conclusions

(1) Autochthonous components, mainly produced by the vital activities of aquatic organisms and bacteria, dominated DOM in the surface sediment of Liangzi Lake. The DOM in the surface sediments of Baoan Lake and Qingshan Lake had weak humification and moderate autochthonous characteristics. DOM humification in the sediment cores of Liangzi Lake, Baoan Lake, and Qingshan Lake gradually decreased from depth to the surface. DOM in the surface sediment of Daye Lake had strong humification and moderate autochthonous characteristics.
(2) In general, the DOM of lake sediments in southeastern Hubei Province has dual-contribution characteristics representing terrigenous and endogenous sources. Cyanobacteria decomposition after death may be the main endogenous DOM source in algal lakes with high nutrient levels. However, the endogenous source DOM in grass lakes with low nutrient levels may mainly come from aquatic plant residues. Therefore, both internal and terrestrial pollution control should be considered in lake ecological restoration and treatment. At the same time, It is noteworthy that the potential internal pollution is caused by cyanobacteria depletion and aquatic residues. Regularly harvest aquatic plants and take appropriate measures to remove algae.

## Figures and Tables

**Table 1:** Basic information of typical lakes in southeastern Hubei.

Lake (Code)	Center of Lake Location (Longitude and Latitude)	Depth of Water (m)	Transparency (cm)	Sample Size	Position of Sediment Core
Liangzi Lake (L)	114°32′47″ E, 30°14′53″ N	2.9 ± 0.5	78 ± 25	12	L1, L9
Baoan Lake (B)	114°43′13″ E, 30°14′38″ N	2.5 ± 0.5	154 ± 83	6	B1, B4
Daye Lake (D)	115°5′46″ E, 30°5′25″ N	3.7 ± 0.3	47 ± 5	15	D3, D11
Qingshan Lake (Q)	115°3′54″ E, 30°14′18″ N	1.5 ± 0.2	51 ± 3	9	Q2, Q8

**Table 2:** Physiochemical indicators of water bodies and surface sediments.

Lake	Water			Surface Sediments		
	TN (mg/L)	TP (mg/L)	COD_Mn_ (mg/L)	TN (mg/kg)	TP (mg/kg)	OM (mg/kg)
L	0.45 ± 0.31	0.09 ± 0.02	3.94 ± 1.56	882.92 ± 275.13	420.63 ± 106.99	41.03 ± 17.97
B	0.71 ± 0.31	0.10 ± 0.04	4.63 ± 0.64	1257.54 ± 313.29	478.80 ± 71.52	58.95 ± 8.76
D	1.45 ± 0.23	0.12 ± 0.03	5.50 ± 0.41	980.78 ± 285.87	638.31 ± 113.71	36.15 ± 10.73
Q	2.90 ± 0.35	0.16 ± 0.03	6.33 ± 0.72	620.74 ± 515.30	1201.77 ± 320.50	71.32 ± 19.09

Note: all data above are “mean ± standard deviation”.

**Table 3:** Characteristics of the three fluorescent components in sediments.

Component	Ex_max_/Em_max_	Conventional Peak Group [37,38]	Description	Matched References
C1	335/422	C	Visible humic-like	[25,30,31,32,39,40]
C2	280,395/473	A, D	UV humic-like, soil fulvic acid	[40]
C3	290/344	T	Protein-like, autochthonous	[30,33,34,35,36,39,41,42]

**Table 4:** Spectral parameters of DOM in surface sediments of different study areas.

Study Area	HIX		BIX		FI		References
	Mean	Range	Mean	Range	Mean	Range	
Nanfei River	4.96	3.87~8.71	0.85	0.66~0.94	2.32	2.23~2.47	[62]
Wuliangsuhai Lake	—	—	—	—	—	1.74~1.96	[53]
Danjiangkou Reservoir	3.72	0.22~7.68	0.74	0.56~0.96	1.71	1.52~2.02	[63]
Lihu Lake	3.41	2.62~4.39	0.77	0.69~0.94	2.05	1.96~2.22	[64]
Jinpen Reservoir	6.03	3.76~7.37	0.73	0.50~0.81	1.72	1.62~1.88	[2]
Konsfjord in the Arctic	2.27	1.66~2.82	0.73	0.64~0.80	1.78	1.76~1.82	[65]
Xiaojia River	3.61	1.77~5.67	0.61	0.53~0.71	1.33	1.16~1.51	[66]
Chaohu Lake	—	—	0.87	0.31~1.54	3.77	2.56~4.89	[15]
Liangzi Lake	1.10	0.40~2.63	2.13	0.78~4.17	1.84	1.73~1.95	This paper
Baoan Lake	2.56	2.22~2.96	0.79	0.73~0.85	1.82	1.75~1.87	This paper
Daye Lake	4.07	0.71~6.24	0.77	0.70~0.92	1.74	1.63~1.80	This paper
Qingshan Lake	2.49	1.36~3.45	0.79	0.74~0.89	1.82	1.75~1.88	This paper

“—” denotes the absence of relevant data in the papers.

## Data Availability

The datasets generated and/or analyzed during the current study are available from the corresponding author on request.

## Supplementary Materials

The following supporting information can be downloaded at: https://www.mdpi.com/article/10.3390/ijerph19127402/s1, Figure S1: Spatial distributions of *a*_350_ in the surface sediments of four typical lakes; Figure S2: Spatial distributions of M values in surface sediments of four typical lakes; Figure S3: Vertical distributions of *a*_350_ in sediment cores from four typical lakes; Figure S4: Vertical distributions of M in sediment cores of four typical lakes; Table S1: Vertical distribution of FDOM in sediment cores; Table S2: Correlations among *a*_350_, TN, TP, OM, and FDOM components; Table S3: Vertical distributions of HIX, BIX, and FI in sediment cores from four typical lakes.
