# Supplementary material for: Characteristics of Dissolved Organic Matter in Sediments of Typical Lakes in Southeastern Hubei Province, China
Source: Int J Environ Res Public Health. 2022 Jun 16;19(12):7402. doi: 10.3390/ijerph19127402 (PMC9223524; doi:10.3390/ijerph19127402)
Supplement: Supplementary file 1 [file ijerph-19-07402-s001.zip › ijerph-1741827-supplementary.pdf]

Characteristics of dissolved organic matter in sediments of typical lakes in southeastern Hubei Province, China

— Supplementary Materials —

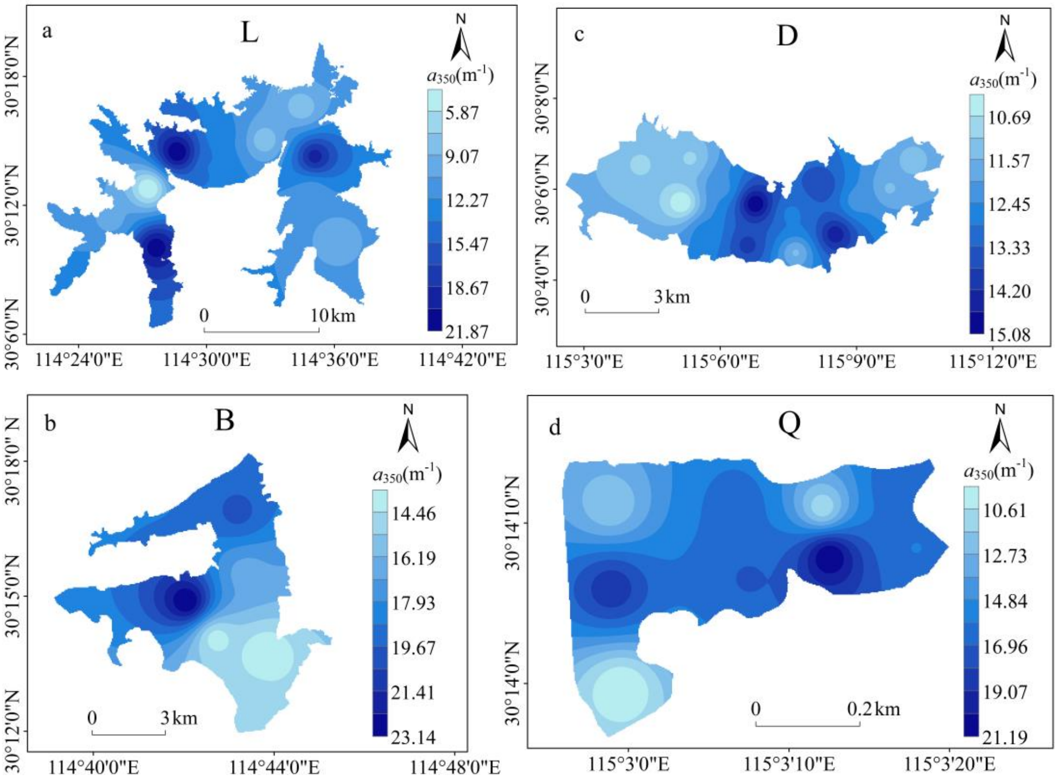

Supplementary Figure S1. Spatial distributions of a_{350} in the surface sediments of four typical lakes.

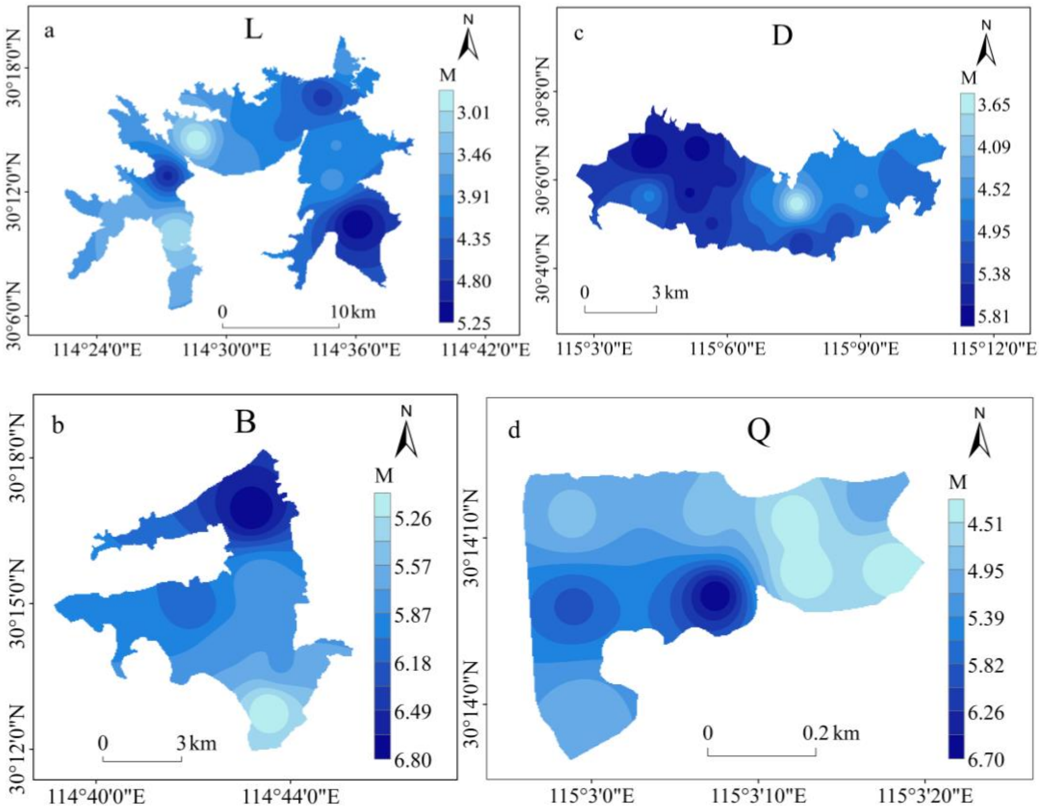

Supplementary Figure S2. Spatial distributions of M values in surface sediments of four typical lakes.

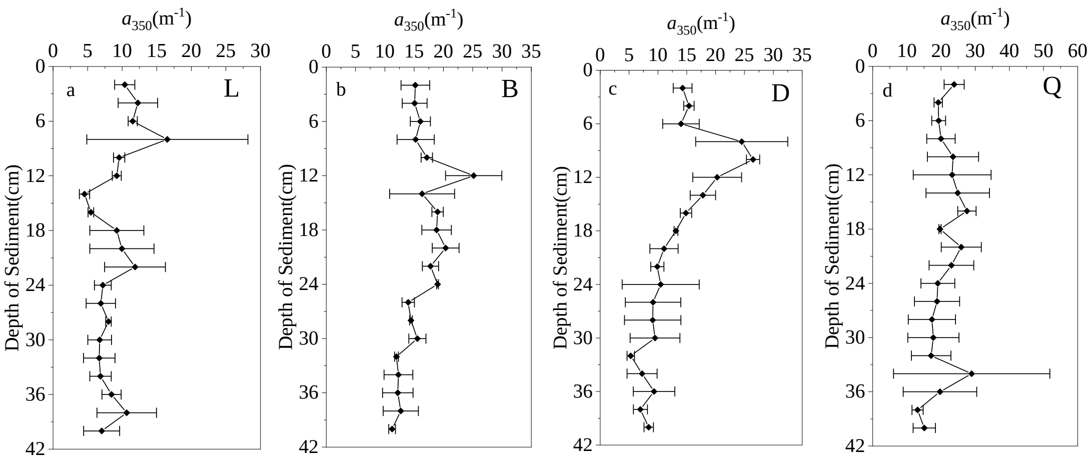

Supplementary Figure S3. Vertical distributions of a_{350} in sediment cores from four typical lakes.

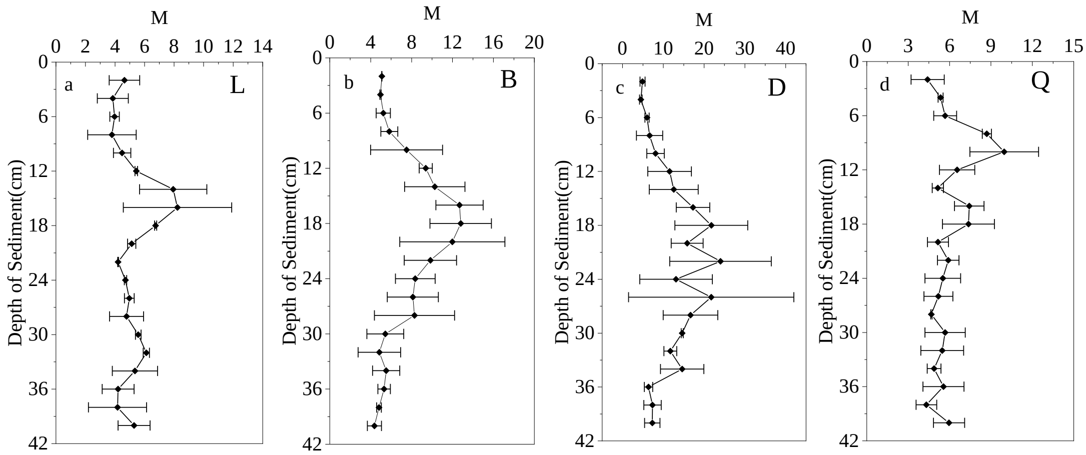

Supplementary Figure S4. Vertical distributions of M in sediment cores of four typical lakes.

Supplementary Table S1. Vertical distribution of FDOM in sediment cores.

Lake	Depth (cm)	F_{1max}(RU)		F_{2max}(RU)		F_{3max}(RU)	
		Min ~ Max	Mean ± S.D.	Min ~ Max	Mean ± S.D.	Min ~ Max	Mean ± S.D.
L	0 ~ 10	0.24 ~ 0.32	0.27 ± 0.03	0.13 ~ 0.20	0.16 ± 0.02	0.11 ~ 0.34	0.16 ± 0.07
	11 ~ 20	0.16 ~ 0.27	0.20 ± 0.04	0.10 ~ 0.17	0.12 ± 0.02	0.08 ~ 0.41	0.18 ± 0.12
	21 ~ 30	0.18 ~ 0.39	0.25 ± 0.08	0.10 ~ 0.21	0.14 ± 0.04	0.07 ~ 0.58	0.21 ± 0.15
	31 ~ 40	0.17 ~ 0.54	0.31 ± 0.14	0.09 ~ 0.26	0.16 ± 0.06	0.06 ~ 0.34	0.21 ± 0.11
B	0 ~ 10	0.39 ~ 0.66	0.56 ± 0.09	0.26 ~ 0.45	0.38 ± 0.06	0.19 ~ 0.34	0.27 ± 0.05
	11 ~ 20	0.30 ~ 0.74	0.51 ± 0.11	0.23 ~ 0.53	0.39 ± 0.09	0.13 ~ 0.33	0.20 ± 0.06
	21 ~ 30	0.27 ~ 0.66	0.45 ± 0.11	0.20 ~ 0.40	0.31 ± 0.06	0.10 ~ 0.20	0.15 ± 0.03
	31 ~ 40	0.20 ~ 0.49	0.37 ± 0.09	0.13 ~ 0.32	0.24 ± 0.06	0.07 ~ 0.17	0.12 ± 0.03
D	0 ~ 10	0.30 ~ 0.62	0.46 ± 0.09	0.21 ~ 0.75	0.42 ± 0.18	0.11 ~ 0.23	0.17 ± 0.04
	11 ~ 20	0.28 ~ 0.54	0.36 ± 0.09	0.20 ~ 0.58	0.34 ± 0.11	0.09 ~ 0.18	0.13 ± 0.03
	21 ~ 30	0.19 ~ 0.33	0.27 ± 0.05	0.11 ~ 0.26	0.19 ± 0.07	0.06 ~ 0.14	0.09 ± 0.03
	31 ~ 40	0.15 ~ 0.34	0.22 ± 0.07	0.08 ~ 0.19	0.13 ± 0.04	0.05 ~ 0.12	0.08 ± 0.03
Q	0 ~ 10	0.53 ~ 0.88	0.73 ± 0.13	0.32 ~ 0.61	0.46 ± 0.09	0.33 ~ 0.61	0.52 ± 0.09
	11 ~ 20	0.40 ~ 0.89	0.65 ± 0.14	0.24 ~ 0.68	0.49 ± 0.13	0.19 ~ 0.50	0.31 ± 0.11
	21 ~ 30	0.43 ~ 0.79	0.64 ± 0.12	0.28 ~ 0.57	0.44 ± 0.10	0.14 ~ 0.29	0.21 ± 0.05
	31 ~ 40	0.44 ~ 1.13	0.66 ± 0.22	0.27 ~ 1.04	0.48 ± 0.24	0.12 ~ 0.49	0.22 ± 0.11

Note: Min, max, and mean denote the minimum, maximum, and mean values, respectively; S.D. denotes standard deviation.

Supplementary Table S2. Correlations among a_{350}, TN, TP, OM, and FDOM components.

		a_{350}	TN	TP	OM	C1	C2	C3
L	a_{350}	1						
	TN	0.141	1					
	TP	0.223	0.388**	1				
	OM	0.309*	0.409**	0.319*	1			
	C1	0.343*	-0.138	0.068	0.229	1		
	C2	0.518**	-0.106	0.063	0.247	0.933**	1	
	C3	0.344*	-0.252	-0.170	0.051	0.121	0.214	1
B		a_{350}	TN	TP	OM	C1	C2	C3
	a_{350}	1						
	TN	0.430**	1					
	TP	0.635**	0.28	1				
	OM	0.507**	0.796**	0.431**	1			
	C1	0.598**	0.371*	0.323*	0.611**	1		
	C2	0.728**	0.363*	0.542**	0.591**	0.931**	1	
	C3	0.591**	0.467**	0.542**	0.707**	0.889**	0.871**	1
D		a_{350}	TN	TP	OM	C1	C2	C3
	a_{350}	1						
	TN	0.609**	1					
	TP	0.294*	0.436**	1				
	OM	0.602**	0.846**	0.333*	1			
	C1	0.653**	0.775**	0.556**	0.808**	1		
	C2	0.918**	0.766**	0.387**	0.787**	0.844**	1	
	C3	0.827**	0.730**	0.534**	0.707**	0.874**	0.885**	1
Q		a_{350}	TN	TP	OM	C1	C2	C3
	a_{350}	1						
	TN	0.274	1					
	TP	0.428**	0.087	1				
	OM	0.476**	0.132	0.528**	1			
	C1	0.815**	0.388**	0.367**	0.475**	1		
	C2	0.911**	0.296*	0.375**	0.449**	0.929**	1	
	C3	0.485**	0.364*	0.368**	0.368**	0.612**	0.421**	1

Note: ** denotes significant correlations at the 0.01 level (two-sided), * denotes significant correlations at the 0.05 level (two-sided).

Supplementary Table S3. Vertical distributions of HIX, BIX and FI in sediment cores from four typical lakes.

Lake	Depth (cm)	HIX		BIX		FI	
		Min ~ Max	Mean ± S.D.	Min ~ Max	Mean ± S.D.	Min ~ Max	Mean ± S.D.
L	0 ~ 10	0.48 ~ 1.95	1.52 ± 0.43	0.84 ~ 3.09	1.21 ± 0.69	1.83 ~ 2.03	1.89 ± 0.07
	11 ~ 20	0.35 ~ 4.70	1.84 ± 1.40	0.97 ~ 4.36	1.88 ± 1.22	1.71 ~ 1.99	1.83 ± 0.09
	21 ~ 30	1.20 ~ 4.47	2.22 ± 1.01	1.06 ~ 1.83	1.39 ± 0.23	1.65 ~ 1.81	1.72 ± 0.05
	31 ~ 40	1.24 ~ 5.97	3.25 ± 1.86	1.06 ~ 1.96	1.33 ± 0.32	1.63 ~ 1.78	1.73 ± 0.05
B	0 ~ 10	2.08 ~ 3.35	2.51 ± 0.40	0.74 ~ 0.91	0.80 ± 0.05	1.73 ~ 1.83	1.79 ± 0.03
	11 ~ 20	2.20 ~ 4.18	3.07 ± 0.64	0.81 ~ 1.05	0.91 ± 0.07	1.66 ~ 1.84	1.73 ± 0.06
	21 ~ 30	2.54 ~ 4.10	3.39 ± 0.49	0.78 ~ 1.02	0.90 ± 0.07	1.61 ~ 1.72	1.66 ± 0.03
	31 ~ 40	2.11 ~ 4.00	3.44 ± 0.61	0.86 ~ 0.99	0.92 ± 0.04	1.58 ~ 1.66	1.63 ± 0.03
D	0 ~ 10	2.61 ~ 4.00	3.15 ± 0.43	0.78 ~ 1.05	0.91 ± 0.11	1.71 ~ 2.09	1.85 ± 0.13
	11 ~ 20	3.14 ~ 3.91	3.59 ± 0.25	0.93 ~ 1.03	0.98 ± 0.03	1.78 ~ 2.01	1.86 ± 0.06
	21 ~ 30	2.93 ~ 4.41	3.44 ± 0.53	0.92 ~ 1.08	1.03 ± 0.05	1.71 ~ 1.90	1.77 ± 0.05
	31 ~ 40	2.50 ~ 4.02	3.15 ± 0.47	0.81 ~ 1.12	1.01 ± 0.09	1.69 ~ 1.81	1.75 ± 0.05
Q	0 ~ 10	0.99 ~ 1.82	1.34 ± 0.25	0.77 ~ 1.14	0.88 ± 0.11	1.77 ~ 1.97	1.86 ± 0.05
	11 ~ 20	1.27 ~ 3.94	2.73 ± 0.96	0.88 ~ 1.15	0.98 ± 0.07	1.72 ~ 2.02	1.85 ± 0.11
	21 ~ 30	3.21 ~ 6.02	4.27 ± 0.91	0.83 ~ 0.95	0.89 ± 0.04	1.69 ~ 1.89	1.76 ± 0.06
	31 ~ 40	2.74 ~ 5.03	4.22 ± 0.65	0.76 ~ 1.00	0.88 ± 0.08	1.66 ~ 1.88	1.74 ± 0.07

Note: Min, max, and mean denote the minimum, maximum, and mean values, respectively; S.D. denotes standard deviation.
